# Broadband wireless communication with space-time-varying polarization-converting metasurface

**DOI:** 10.1515/nanoph-2023-0027

**Published:** 2023-03-07

**Authors:** Qi Hu, Ke Chen, Yilin Zheng, Zhiyuan Xu, Jianmin Zhao, Jian Wang, Yijun Feng

**Affiliations:** School of Electronic Science and Engineering, Nanjing University, Nanjing, 210093, China

**Keywords:** broadband, reconfigurable metasurfaces, space-time-varying, wireless communication

## Abstract

Reconfigurable metasurfaces have emerged as a promising alternative to the conventional transmitter of wireless communication systems, due to their abilities of encoding digital information onto electromagnetic properties without complex radio-frequency chains. However, most of them are still limited to narrow operation bandwidth. Here, we propose a broadband metasurface-based wireless communication system that can actively adapt to multiple users located at versatile directions through joint modulation of digital signals in the time domain and wave scatterings in the space domain. As exemplary demonstrations, highly directive beams are generated to enhance regional signals in real-time customized for users in desired directions and reduce the signal leakage in undesired directions. Experiments are carried out to verify that the system can provide stable wireless communication service in a broad band of 3.7–5.1 GHz, within which the transmitted color picture enabled by the time-varying spatial modulation of metasurface can be successfully recovered at the user terminals. The proposed system may offer untapped potentials for next-generation communications and radar systems where regional signal enhancement, active adaption to users, and large channel capacities are required.

## 1 Introduction

During the past decades, metamaterials have attracted great interest from scientific researches to engineering fields due to their artificially engineered structures with unique electromagnetic (EM) properties that do not exist in nature, which have provided unconventional abilities of manipulating EM waves in subwavelength scales. Metasurfaces, as the two-dimensional (2D) equivalence of metamaterials, not only reserve the unprecedented wave manipulation abilities, but also possess considerable strengths of low profiles, easy fabrication, and compact configuration. Therefore, metasurfaces have enacted diverse functional devices, such as flat meta-lenses [1, 2], invisibility cloaks [3], and holographic imagers [4–6]. To overcome restrictions of fixed performances after the fabrication, reconfigurable metasurfaces are proposed to enable real-time dynamic control of EM waves by integrating active components, such as positive-intrinsic-negative (PIN) diodes [7], varactors [8], liquid crystals [9], and micro-electromechanical systems [10, 11]. It has been verified that by introducing field discontinuities across the interface, finite types of meta-atoms can be utilized to tailor EM wavefronts and can be further abstracted to digital coding symbols [12]. Thus, by adopting versatile spatial coding distributions through varying the external stimulation by means of software-driven hardware controllers, for example, field-programmable gate arrays (FPGAs), metasurfaces can evolve into programmable fashions. Programmable metasurfaces can provide flexible tuning among different functions and thus facilitating a variety of intriguing applications, such as dynamic beamforming [13–15], reprogrammable imaging [16], and wireless communications [17, 18]. For instance, by synergizing metallic patterns and PIN diodes in sandwich meta-structures, a programmable metasurface can successfully implement various functions, including anomalous deflections, orbital angular momentum (OAM) generation, and beam focusing in polarization-multiplexed channels [19].

As one of the frontiers of real-world applications, programmable metasurfaces exhibit great potentials and compatibilities in the field of wireless communications through manipulating the wave-information-matter interactions [20]. On one hand, since the users in remote areas generally suffer from poor wireless communication quality due to the severe channel fading and blocking obstacles, programmable metasurfaces can be deployed to dynamically adjust EM wave propagation and reflect/refract the signals towards user terminals, thus enhancing the regional signals and the communication service [21–23]. On the other hand, programmable metasurfaces have emerged as a promising alternative to the conventional transmitter of wireless communication systems, because their reflection and transmission properties can be digitally modulated in a time-varying manner, thus making them a natural information transmitter without additional complex radio-frequency (RF) chains including oscillators, nonlinear mixers, and wideband power amplifiers [24, 25]. So far, many efforts have been devoted to implementing metasurface-based wireless communication systems by directly encoding the digital information onto EM waves. For example, modulation schemes of binary frequency-shift keying (BFSK), quadrate phase-shift keying (QPSK) and quadrate amplitude modulation (QAM) have been demonstrated by modifying the EM properties of the metasurfaces through altering the duty ratios and time delays of the time-varying modulation signals in square waveforms [26, 27]. Despite these significant progresses, most of them only encode the transmitted information in the time domain, leaving space-domain resources unexploited to enable practical functions, for example, dynamic communication service coverage for multi-users or moving users; moreover, the operation bandwidth may be further improved as the large channel capacities are increasingly demanded in the next-generation communications [28].

To surmount aforementioned restrictions, here we propose a broadband metasurface-based wireless communication system that can actively adapt to users located in versatile directions through joint modulation of digital signals and far-field wave scatterings. To be specific, the reconfigurable metasurface is designed with a phase difference of 180° between the cross-polarized reflections in a broad frequency band from 3.7 to 5.1 GHz. It is achieved by modulating the inserted PIN diodes and can be further utilized to enable the joint modulation of polarizations, phases, and amplitudes. Versatile sets of space-time-varying coding patterns are calculated to generate dual or multiple highly directive beams pointing to desired directions with different digital information, not only resulting in enhancement of regional signals and reduction of signal leakage in undesired directions, but also customized to simultaneously offer reliable communication services to user terminals. Moreover, by modulating the mapping relationships between binary symbols of bit streams and the space-time-varying coding patterns, the system can actively adapt to users located at various directions through beam steering. As the experiment verifications, a color picture is directly transmitted and successfully recovered at user terminals.

## 2 Results

**Figure 1:**
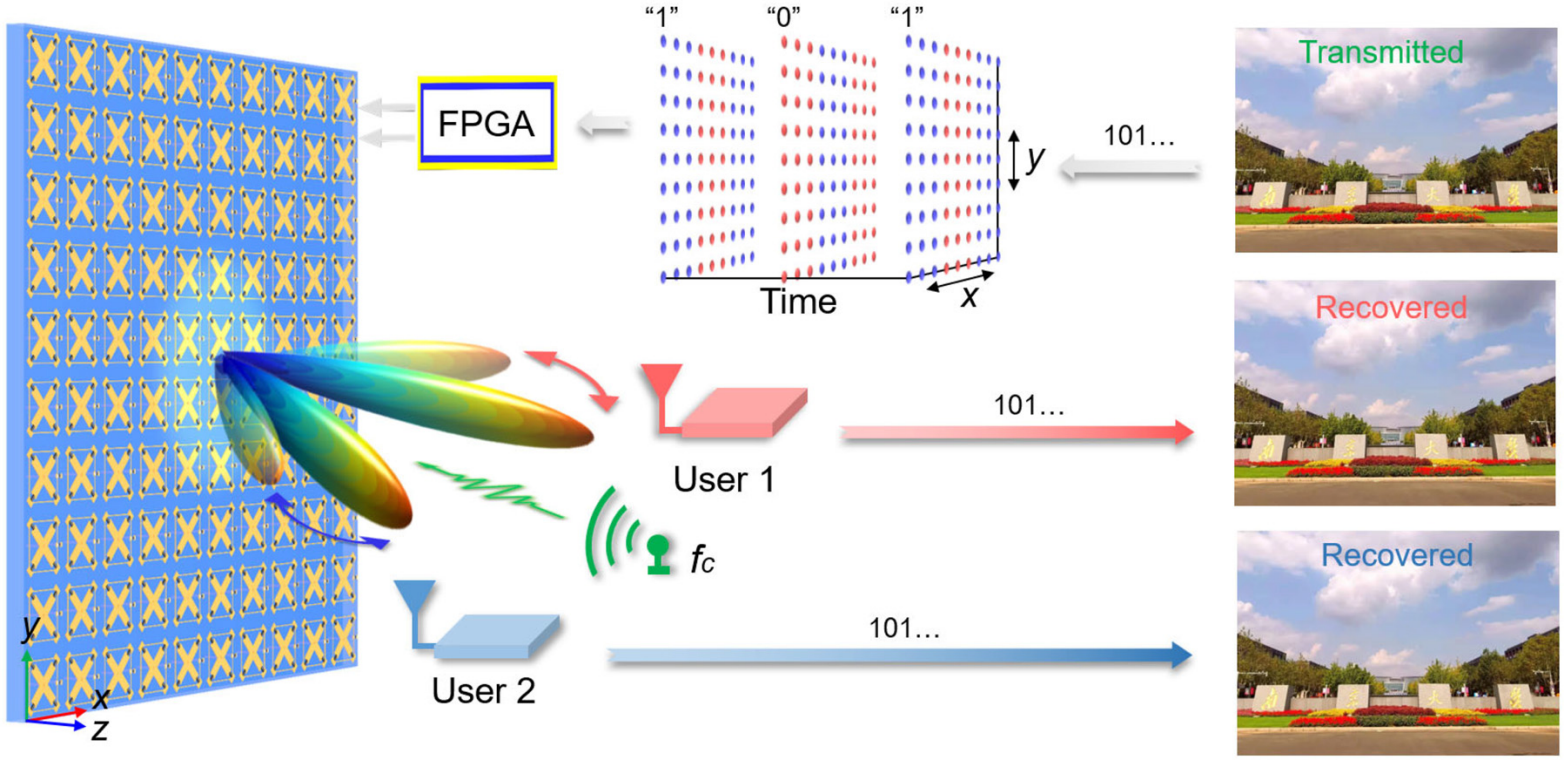
Conceptual illustration of the broadband wireless communication system with beam-steerable capability based on the space-time-varying metasurface. By modulating the metasurface with time-varying spatial patterns translated by the transmitted information, highly directive beams are simultaneously generated to enhance regional signals and reduce signal leakage in undesired directions, customized for time-division multiuser schemes.

As depicted in Figure 1, the broadband RF-chain-free wireless communication system with beam-steerable capability consists of a signal source, a reconfigurable metasurface, multiple users, and an FPGA-based computer-controlled system, in which the original information is synchronously transmitted and successfully recovered at multiple users in different directions. During the information transmission, firstly, the original information, for example, a color picture, is interpreted into a bit stream composed of binary symbols of “0” and “1”, for example, “101 … ”. Then, the bit stream is mapping to space-time-varying coding patterns that are utilized as the controlling signals to modulate the proposed metasurface through the hardware control system. Under the illumination of a carrier wave emitted by the signal source, the reflected wave from the metasurface not only carries original information but also radiates in different pre-designed directions, customized for time-division multiuser schemes. Ultimately, by judging the phases of the signals at user sides, bit streams can be successfully recovered and further translated into a color picture. We note that there are two key steps to implement aforementioned RF-chain-free wireless communication system. The first step is to establish the relationship between the binary symbols representing digital information and the space-time-varying coding patterns utilized to generate highly directive beams. The second one is to design a metasurface that supports the dynamic phase control in a broad frequency band.

### 2.1 Design principle

Assigning the monochromatic EM wave incidence of frequency *f*_*c*_ as *E*_*i*_(*t*) = e^*j*2π*fct*^ and assuming the modulation speed of the metasurface much smaller than the incident frequency, the far-field scattering pattern from a space-time-varying metasurface consisting of *M* × *N* meta-atoms can be derived as [29]:

(1) (1) $$\begin{array}{ll} f\left(\theta ,\varphi ,t\right) & =\sum_{n=1}^{N}\sum_{m=1}^{M}E_{mn}\left(\theta ,\varphi \right)R_{mn}\left(t\right)\\ & \;\times \exp\left\{j\frac{2\pi }{\lambda_{c}}\left[\left(m-1\right)d_{x}\sin\theta \cos\varphi \right.\right.\\ & \;+\left.\left.\left(n-1\right)d_{y}\sin\theta \sin\varphi \right]\right\}, \end{array}$$

where *E*_*mn*_(*θ*, *φ*) is the far-field pattern of the (*m*, *n*)th meta-atom, *θ* and *φ* are the elevation and azimuth angles respectively, *R*_*mn*_(*t*) is the time-varying reflection property of the (*m*, *n*)th meta-atom, *λ*_*c*_ is the wavelength of incidence, *d*_*x*_ and *d*_*y*_ are the periods of the meta-atom along the *x*- and *y*-directions. The above equation indicates that the far-field scattering pattern of a metasurface can be possibly engineered by properly tuning the phase profiles on its aperture. It is found that even with meta-atoms of binary tunable phase responses, versatile beam functionalities including beam scanning and multi-beam generation can be realized in a dynamic manner [30]. Hence, phase-tunable metasurfaces could provide the dynamic reshaping of the incidence into desired user locations in the wireless communications scheme.

On the other hand, encoding digital information onto the carrier wave relies on the change of the wave behaviors that should be captured with difference at different time moments when it is observed at the receiving terminal. It is obvious that an arbitrary phase difference *φ*_0_ between two far-field scattering patterns pointing to same directions at two moments of *t*_0_ and *t*_1_, can be directly obtained by attaching an initial phase *φ*_0_ to each meta-atom, irrespective of the metasurface spatial profiles, which can be expressed as

(2) $$R_{mn}\left(t_{1}\right)=R_{mn}\left(t_{0}\right)e^{j\varphi 0}\Rightarrow f\left(\theta ,\varphi ,t_{1}\right)=f\left(\theta ,\varphi ,t_{0}\right)e^{j\varphi 0}.$$

If the proposed reconfigurable metasurface meta-atom has two switchable reflections of *R*_0_ = *A*e^*j*0^ and *R*_1_ = *A*e^*j*π^, then a 180° phase difference of the scattering wave in the far-field region detected at *t*_0_ and *t*_1_ can be directly achieved as all meta-atoms flipped at *t*_1_, providing an efficient tool to encode the transmitted information. Moreover, such phase flipping with a 180° phase will not affect the beam directions and thus the signal quality at the user sides. Therefore, by switching the space-time-varying coding patterns, the wave scattering manipulation in the space domain and the digital information modulation in the time domain can be simultaneously realized.

### 2.2 Metasurface design

**Figure 2:**
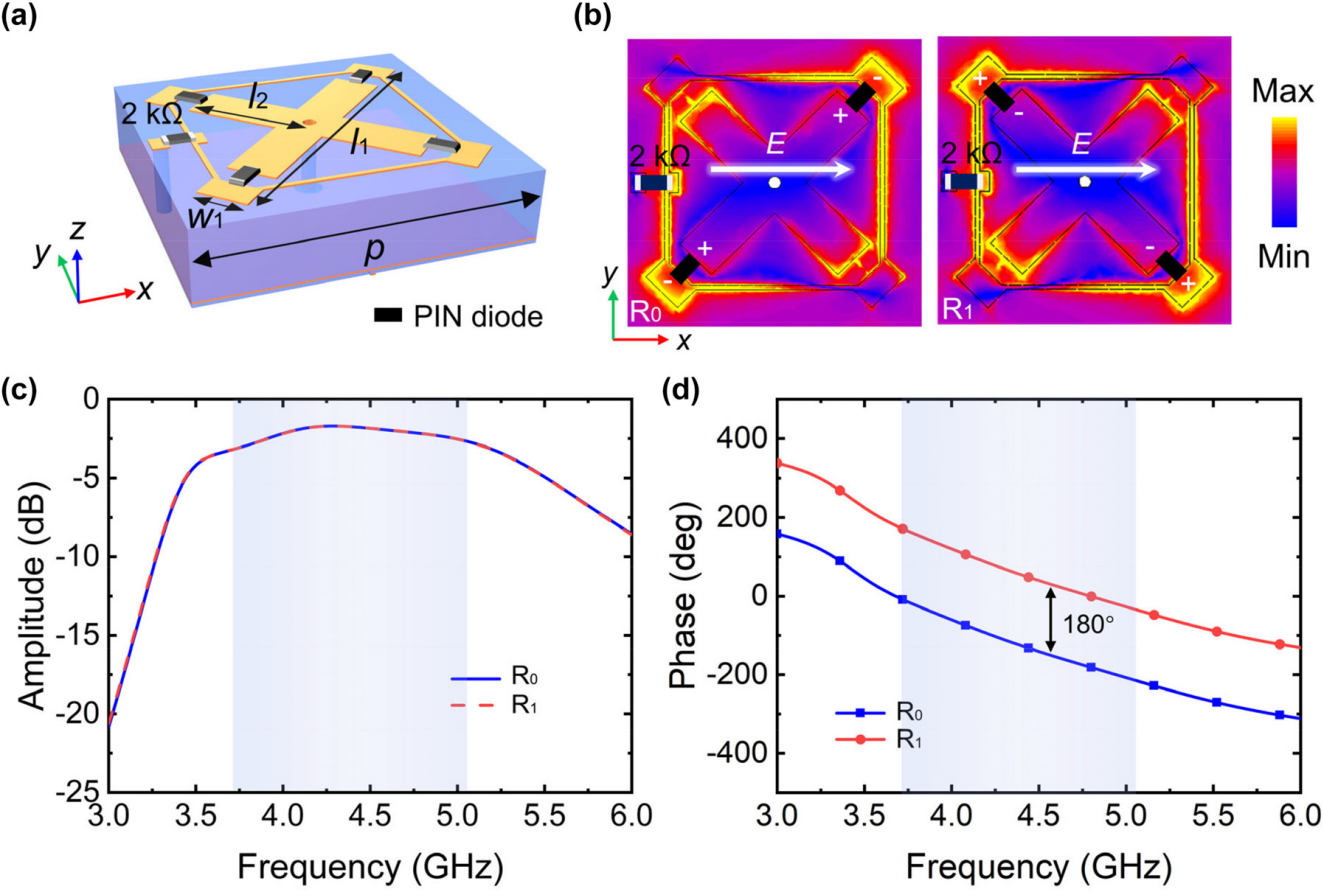
The proposed meta-atom and its simulated cross-polarized performances. (a) Schematic of the proposed meta-atom. (b) The electric field distribution of the meta-atom operating as “R_0_” and “R_1_” at 4.4 GHz. Simulated cross-polarized (c) reflection amplitude and (d) reflection phase of the proposed meta-atom.

To validate our modulation strategy and implement the metasurface-based wireless communication system, a polarization-converting metasurface supporting dynamic broadband phase control is designed. As illustrated in Figure 2(a), the proposed meta-atom has a multilayer configuration of two substrate layers sandwiched by three metallic layers and separated at a distance of 3.8 mm and 0.2 mm. The top metallic layer is composed of two cross-rectangular patches connected by a grid texture, and each of them is symmetrically cut off and connected by a PIN diode. The PIN diodes are generally embedded to the position where the electric field is mainly concentrated to maximally interact with the meta-structure. Meanwhile, a resistor of 2 kΩ is inserted in the grid texture to choke the RF leakage. The second metallic layer, connected to the top cross-rectangular patterns by a metallic via hole, not only functions as one of the electrodes of biasing voltages, but also operates as a ground plane to block the transmission. The third metallic layer functions as the other electrode of biasing voltages. The dielectric layer is made of F4B with relative permittivity of 4.3 and loss tangent of 0.0035. Metallic layers are made of copper with a thickness of 0.018 mm and a conductivity of 5.8 × 10^7^ S/m. PIN diodes (Skyworks, SMP1345-079LF) are employed as the active components to enable tunable EM performances, which can be equivalently modeled as a resistor–inductor–capacitor (RLC) series connection circuit with parameters *R* = 50 Ω, *C* = 0.13 pF when switched off and *R* = 1.5 Ω, *L* = 0.7 nH when switched on.

To further investigate the EM performances of the metasurface design, full-wave simulations are performed using the commercial software CST Microwave Studio, and periodic boundary condition is employed in *x*- and *y*-direction while open boundary condition for *z*-direction. We note that the two PIN diodes along the 45°-direction [ $\sqrt{2}/2\left(\vec{e_{x}}+\vec{e_{y}}\right)$ ] or 135°-direction [ $\sqrt{2}/2\left(-\vec{e_{x}}+\vec{e_{y}}\right)$ ] are simultaneously modulated to operate at identical states. Moreover, the PIN diodes loaded along orthogonal directions always operate at opposite states to excite inversed currents. We encode the operation state of switching on the diodes along the 45°-direction and switching off the diodes along the 135°-direction as code “R_0_”. On the contrary, the other state is encoded as “R_1_” by switching off the diodes along the 45°-direction and switching on the diodes along the 135°-direction. Figure 2(b) depicts the electric field distributions of the meta-atom operating as “R_0_” and “R_1_” under an *x*-polarized illumination at 4.4 GHz. For a clear view, we mark the switched-on diodes and leave out the switched-off diodes. It is evident that the electric field concentrates on the gap where the diodes are switched off. By alternatively switching the PIN diodes along orthogonal directions, the effective lengths of the crossed patches containing the diodes are directly modulated and thus leading to almost mirror symmetric electric field distribution along *x*-axis. It is such a mirror symmetry of the electric fields (or induced currents) that stem from the structure nature finally leads to the exactly inversed reflection phases between the two working states; see more results in Figure S1 (Supporting Information). As a result, the reflection phase difference is always 180°, regardless of high or low reflection amplitude. Therefore, the optimization object of the meta-atom can be reduced to high cross-polarized reflection amplitude with bandwidth as broad as possible. With optimized geometric parameters of *p* = 13 mm, *l*_1_ = 14 mm, *l*_2_ = 5.2 mm, and *w*_1_ = 2 mm, the simulated cross-polarized reflections of the meta-atom at two operation states under an *x*-polarized illumination are illustrated in Figure 2(c) and (d). We can observe a phase difference of exact 180° between two coding states in the broad frequency range from 3.7 GHz to 5.1 GHz. The remaining energy is almost absorbed due to the intrinsic loss of materials and diodes; see more details in Figure S2 (Supporting Information). Hence, the proposed meta-atom is designed with abilities of joint modulation of polarizations, amplitudes, and phases. Since phase modulation signals have more advantages in anti-jamming capacity than amplitude modulation signals, the digital signal modulation scheme in this work is adopted as phase modulation, which can be used for dynamic beam steering through a proper spatial distribution of the meta-atoms.

## 3 Metasurface-based wireless communication system

Based on the above modulation principles, a metasurface-assisted wireless communication system is constructed to perform experimental verifications. A metasurface sample consisting of 30 × 30 meta-atoms with a total size of 390 × 390 mm^2^ was fabricated using a commercial standard printed circuit board (PCB) technique. For simplicity, here we connect the meta-atoms in the same column (*y*-direction) together, so that meta-atoms in the same column share a common bias voltage and the spatial coding pattern (bias voltage) of the metasurface is reduced to a chain. As the design examples, we aim to simultaneously provide reliable communication service for two users; however, it can be directly extended to more users by adopting optimization algorithms for the generation of multiple highly directive beams.

**Figure 3:**
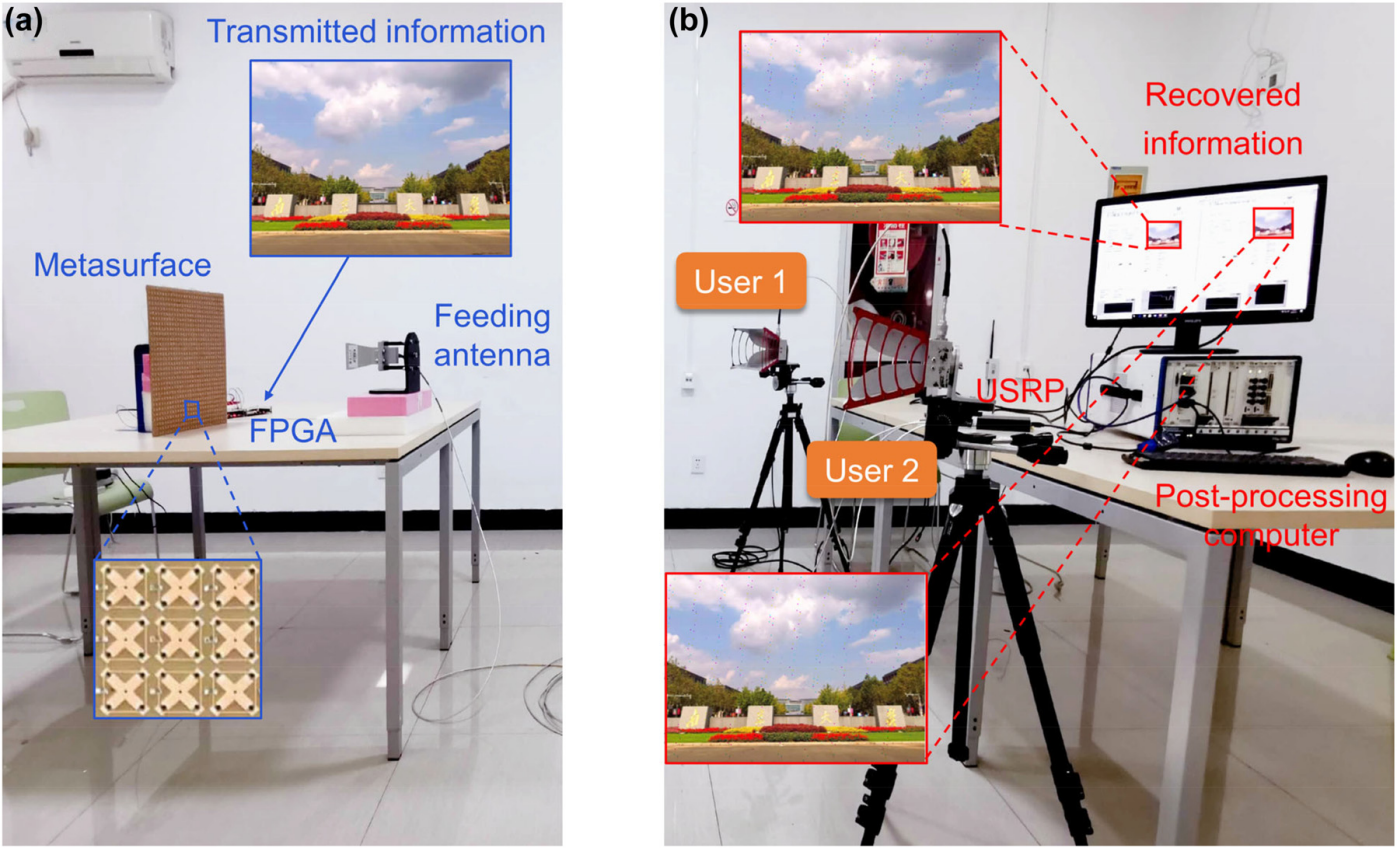
Experimental scenario of the metasurface-based wireless communication system. (a) Transmitting terminal. Insets illustrate the transmitted information and a zoom-in view of the fabricated sample. (b) Receiving terminal. Insets illustrate the recovered information at the two user terminals at 4.6 GHz when binary symbols “0” and “1” are fixed to spatial patterns of “R_0_R_0_R_0_R_0_R_0_R_1_R_1_R_1_R_1_R_1_…” and “R_1_R_1_R_1_R_1_R_1_R_0_R_0_R_0_R_0_R_0_…”, respectively.

As detailed in Figure 3(a), the transmitting terminal is composed of the reconfigurable metasurface, a linearly polarized horn antenna, and a computer-controlled FPGA-based controller. The horn antenna is connected to one port of a universal software radio peripheral (USRP, B210) and utilized to produce the monochromatic carrier wave. The FPGA-based controller is loaded with control signals that carry the transmitted information. The receiving terminal consists of two linearly polarized antennas and a post-processing computer, as shown in Figure 3(b). The two receiving antennas acting as two users located at different directions are connected to two ports of the identical USRP. During wireless communication, the transmitted information at the transmitting terminal, for example, a picture is first translated into bit streams. Then, the bit streams are mapping to space-time-varying coding patterns, which are utilized as the control signals to drive the proposed metasurface through the FPGA-based controller. When the incidence imposes on the metasurface, each meta-atom not only acts as the secondary source to radiate the energy received from the primary incidence into twin beams, but also acts as an information transmitter that enacts different receiving phases at the user terminals, thus enabling the simultaneous modulation of far-field scatterings and digital information. Ultimately, the digital information is de-mapping into binary symbols by judging the reflection phases and then recovered as a picture through the USRP and post-processing computer at the two user terminals.

**Figure 4:**
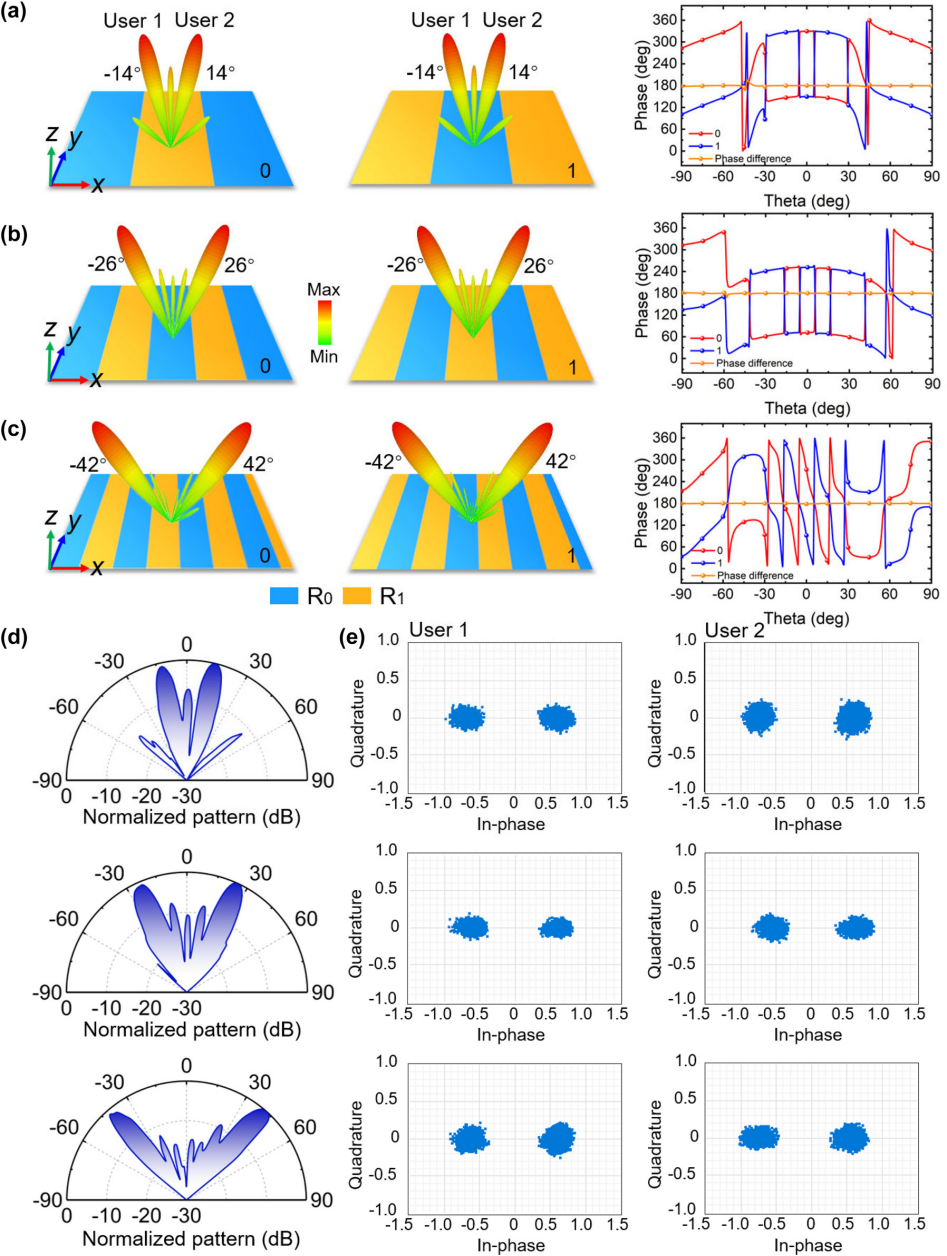
Simulated/measured far-field scattering patterns and measured constellation diagrams with versatile mapping relationships between digital information and spatial coding patterns. Simulated scattering patterns and the phase difference between symbol “0” and “1” corresponding to spatial coding patterns of (a) “R_0_R_0_R_0_R_0_R_0_R_0_R_0_R_0_R_0_R_0_R_1_R_1_R_1_R_1_R_1_R_1_R_1_R_1_R_1_R_1_…” and “R_1_R_1_R_1_R_1_R_1_R_1_R_1_R_1_R_1_R_1_R_0_R_0_R_0_R_0_R_0_R_0_R_0_R_0_R_0_R_0_ … ”, (b) “R_0_R_0_R_0_R_0_R_0_R_0_R_1_R_1_R_1_R_1_R_1_R_1_ … ” and “R_1_R_1_R_1_R_1_R_1_R_1_R_0_R_0_R_0_R_0_R_0_R_0_ … ”, and (c) “R_0_R_0_R_0_R_0_R_1_R_1_R_1_R_1_ … ” and “R_1_R_1_R_1_R_1_R_0_R_0_R_0_R_0_ … ”. (d) Measured far-field results under the modulation of “R_0_R_0_R_0_R_0_R_0_R_0_R_0_R_0_R_0_R_0_R_1_R_1_R_1_R_1_R_1_R_1_R_1_R_1_R_1_R_1_…”, “R_0_R_0_R_0_R_0_R_0_R_0_R_1_R_1_R_1_R_1_R_1_R_1_…”, and “R_0_R_0_R_0_R_0_R_1_R_1_R_1_R_1_…”. (e) Measured constellation diagrams at users located at the main beam directions in aforementioned three cases.

According to generalized Snell’s law, two anomalously reflected beams can be directly generated by arranging the two-state meta-atoms into periodic grating-like distribution, and the elevation angles *θ* of the directive beams can be predicted by *θ* = ±sin^−1^(*λ*_*c*_/Γ) [12]. Here, Γ is the period of the spatial coding pattern and *λ*_*c*_ is the free-space wavelength. Therefore, the proposed metasurface system can adaptively provide communication service for users at versatile directions through beam steering which is simply achieved by modulating the spatial coding patterns. We highlight that in our space-time-varying coding strategy, the binary symbols of “0” and “1” for information transmission are realized by two spatial patterns, which can generate two far-field scatterings with main directive beams of same elevation angles but distinguished by a 180° phase difference. As the illustrative examples, the binary symbols of “0” and “1” are mapping to periodic spatial coding patterns varying along *x*-direction of “R_0_R_0_R_0_R_0_R_0_R_0_R_0_R_0_R_0_R_0_R_1_R_1_R_1_R_1_R_1_R_1_R_1_R_1_R_1_R_1_…” and “R_1_R_1_R_1_R_1_R_1_R_1_R_1_R_1_R_1_R_1_R_0_R_0_R_0_R_0_R_0_R_0_R_0_R_0_R_0_R_0_…”, “R_0_R_0_R_0_R_0_R_0_R_0_R_1_R_1_R_1_R_1_R_1_R_1_…” and “R_1_R_1_R_1_R_1_R_1_R_1_R_0_R_0_R_0_R_0_R_0_R_0_…”, “R_0_R_0_R_0_R_0_R_1_R_1_R_1_R_1_…” and “R_1_R_1_R_1_R_1_R_0_R_0_R_0_R_0_…”, respectively. In these cases, according to theoretical prediction, the incidence is expected to be deflected to anomalous angles of *θ* = ±14°, ±26°, and ±42° in the *x*–*z* plane at 4.6 GHz, with a 180° phase difference at the beam direction between the “0” and “1” symbols in each case. The simulated far-field scattering patterns and the phase difference between the two symbols in each case are displayed in Figure 4(a)–(c), respectively, with open boundary condition for all directions in simulations. It is observed that the reflected beams are symmetrically split into dual beams at the pre-designed deviation angles and the phase difference between two symbols can almost maintain 180° in the full angular range of backward space, satisfying the requirement for information transmission. Such highly directive beams can enhance regional signals and reduce signal leakage in undesired directions, as well as potentially promote information confidentiality and prevent eavesdropping. The far-field scattering patterns are not limited to dual directive beams. A design example of providing simultaneous wireless communication service for three users is detailed in Figure S3 (Supporting Information).

To validate the metasurface design and modulation principle, cross-polarized scattering patterns of the metasurface under the modulation of aforementioned patterns (symbol “0”) in each case are measured in a microwave anechoic chamber, as presented in Figure 4(d). The measured scattering patterns are calibrated to the co-polarized reflections of a same-sized metallic plate. Restricted by the measurement conditions, the measured results only cover the scattering angles ranging from −50° to 50°; nevertheless, it is sufficient to prove the far-field properties of the metasurface as the scattering energy is mostly confined in this angular domain. Under an *x*-polarized incidence, the measured twin beams are approximately pointed to ±14°, ±26°, and ±42°, respectively, which conform well to both theoretical predictions and simulated analysis in each case. Thus, the beam directions can be dynamically tuned by controlling the bias voltages applied onto the metasurface in real-time. Some slight discrepancies in the side-lobes are observed between the simulated and measured results, which are mainly caused by imperfect fabrications and measurements, for example, the little distorted flatness of the prototype. Owing to the increase of beam width at larger beam steering angles, the intensity measured by users at 4.6 GHz with steering angle of ±42° is approximately 1.6 dB lower than that of ±14°, which can cause efficiency loss during information transmission.

Ultimately, direct information transmission is carried out by varying mapping relationship between binary symbols and spatial modulation patterns. We define the user with the negative elevation angle as User 1 and the other one as User 2. By anomalously deflecting the incidence to the directions where the users locate, we simultaneously measure the constellation diagrams of the receiving signals at user terminals. As shown in Figure 4(e), the constellation diagrams measured at the main beam direction are in good consistence with the expectations in each case, where the absolute amplitudes are almost equal but the phases are with a 180° difference for the symbol of “0” and “1”. The transmitted information can be successfully recovered by the users in each case, and two of the recovered pictures for the case of ±26° are displayed in Figure 3(b) as examples, demonstrating that the proposed wireless communication system can provide reliable communication services. Besides phase modulation scheme, wireless communication adopting amplitude modulation scheme is also exemplarily demonstrated to verify the abilities of the proposed metasurface; see more details in Figure S4 (Supporting Information).

**Figure 5:**
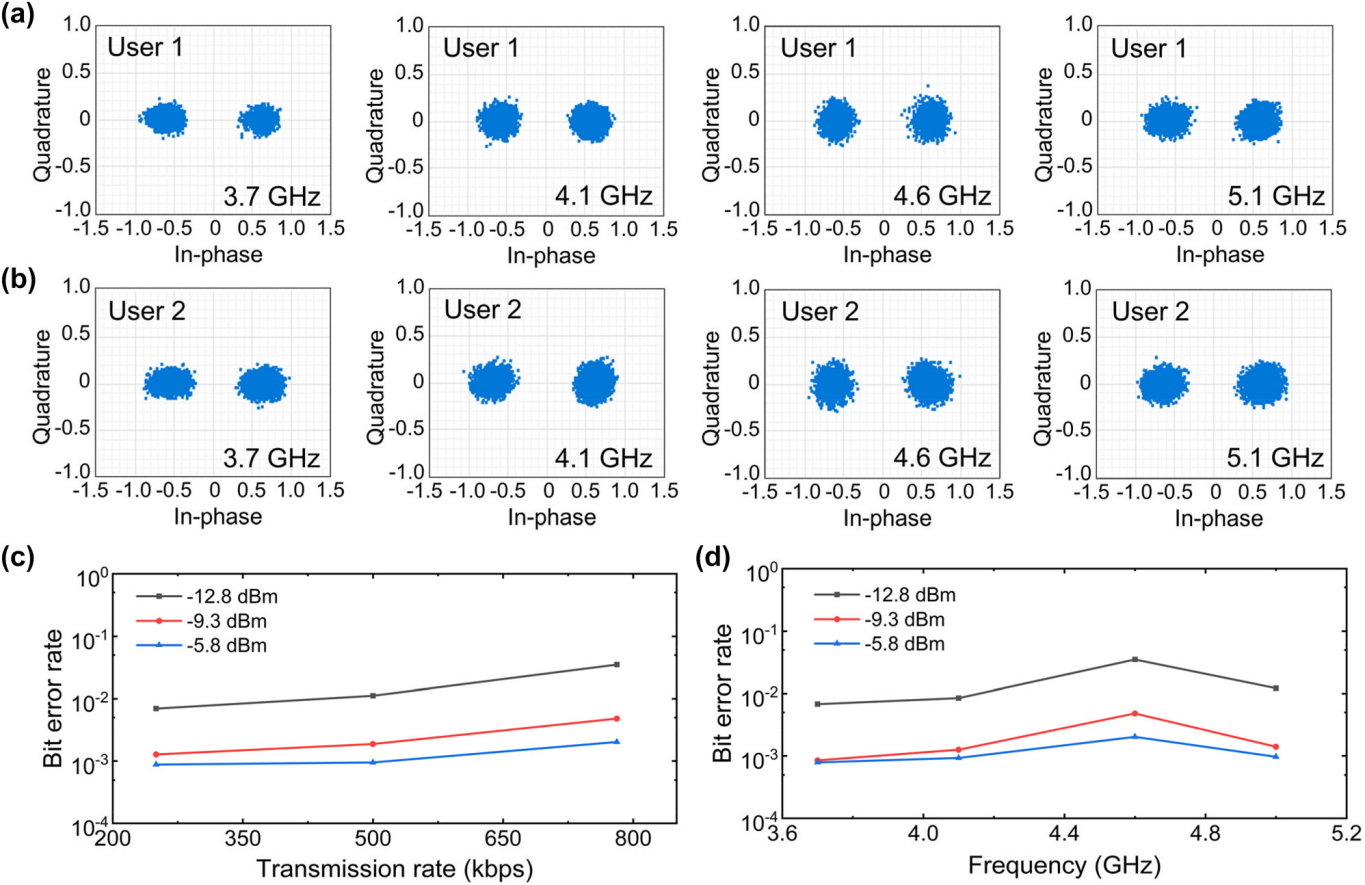
Measured constellation diagrams and BER performances with broadband characteristics. Measured constellation diagrams at (a) User 1, and (b) User 2 at 3.7 GHz, 4.1 GHz, 4.6 GHz, and 5.1 GHz. Measured BER performance (c) as a function of transmitting power and transmission rate at 4.6 GHz, and (d) as a function of transmitting power and operation frequency at maximum transmission rate of 781.25 kbps.

The broadband performances of the proposed metasurface-assisted wireless communication system are also experimentally tested. As the illustrative example, the binary symbols “0” and “1” are fixed to mapping relationships with spatial patterns of “R_0_R_0_R_0_R_0_R_0_R_1_R_1_R_1_R_1_R_1_…” and “R_1_R_1_R_1_R_1_R_1_R_0_R_0_R_0_R_0_R_0_…” varying along the *x*-direction. The performances at several frequencies of 3.7 GHz, 4.1 GHz, 4.6 GHz, and 5.1 GHz are investigated. First, the constellation diagrams measured at the main beam directions at different frequencies are shown in Figure 5(a) and (b). It is observed that the received symbols can be clearly separated to two areas in constellation diagrams at both users, indicating the practicability of our communication system. It should be noted in the experiments, due to the dispersion nature, the maximum beam direction is varying as a function of the operation frequency, analogous to frequency-scanning antennas [31, 32]. Therefore, we may also use this metasurface for frequency-scanning wireless communication, for example, the main beam direction in this case continuously changes from ±40° to ±28° when the frequency increases from 3.7 GHz to 5.1 GHz, and more details can be found in Figures S5 and S6 (Supporting Information). Therefore, without complex optimization procedure, we can get a continuous wide-angle beam range for direct wireless communication by utilizing the combination of frequency-scanning and spatial coding.

As one of the most important indexes of the communication system, the bit error rate (BER) performance is also testified, as a function of the emitting power and the transmission rate. As depicted in Figure 5(c), the BER performances are improved by increasing the emitting power at an illustrative operation frequency of 4.6 GHz. It is reasonable because the constellation diagrams will be more concentrated to two points, as a result of the enhanced signal to noise ratio when the emitting power becomes larger, which finally decreases the possibilities of misjudgment. On the other hand, with the increase of transmission rates, the existence of response time of circuit components and the distortion of modulation signals will generate a higher noise power, thus leading to the deterioration of BER performance. During the information transmission, each message symbol represents log_2_|*M*| bits digital information, where *M* is a set of constellation points with the cardinal number |*M*|. Therefore, in BPSK modulation scheme (*M* = 2), a maximum modulation speed of 781.25 kHz may lead to an upper limit of transmission rate of 781.25 kbps. To demonstrate the broadband properties, BER performances are also testified at several frequencies of 3.7 GHz, 4.1 GHz, 4.6 GHz, and 5.1 GHz, with the maximum transmission rate of 781.25 kbps. As shown in Figure 5(d), BERs can approximately maintain stable across the operation band for a given emitting power. However, when the power is reduced to a certain range, for example, lower than −12.8 dBm, the BER apparently increases as the result of the reduced signal to noise ratio. It is noted that BER may also increase at larger beam steering angles due to the reduced intensity and signal to noise ratio, which can be improved by adopting active components with faster switching speed, promoting the modulation circuit bandwidth, and optimizing the metasurface reflections.

## 4 Conclusions

In summary, we have proposed a broadband metasurface-based wireless communication system with simplified architecture for direct information encoding to enhance regional signals at versatile directions. Through joint modulation of digital signals in the time domain and the wave scatterings in the space domain, the information is directly encoded onto the EM waves by the time-varying change of the spatial phase patterns on the metasurface aperture, without additional RF chains. In the experiments, the transmitted information is successfully recovered at the user terminals. We envision that the phase configurations of the meta-atom could be improved to realize multilevel modulation scheme and therefore to transmit independent information for multiple users located at asymmetric directions. Furthermore, the proposed system could evolve to a self-adaptive version by utilizing sensors (for example, cameras) to capture the locations of users, which may be used in real wireless communications. In that case, the users’ locations are dynamically tested and sent to the post-processing computer, and then the hardware-controller is controlled to output the required voltage signals according to the predefined mapping relationship between binary digital symbols and spatial coding patterns, which finally leads to the information transmission to the users. By adopting alternative active components with faster responses, the architecture may be extended to millimeter wave region or even higher frequencies [33–35]. The proposed approach provides an effective architecture with advantages of simplified structure, enhanced regional signals, large channel capacities, and active adaption to users, which may have potentials in secure communications, radar systems, target tracking, etc.

## Supplementary Material

Supplementary Material Details

## References

[1] Aieta F., Genevet P., Kats M. (2012). Aberration-free ultrathin flat lenses and axicons at telecom wavelengths based on plasmonic metasurfaces. Nano Lett..

[2] Ogawa C., Nakamura S., Aso T., Ikezawa S., Iwami K. (2022). Rotational varifocal moiré metalens made of single-crystal silicon meta-atoms for visible wavelengths. Nanophotonics.

[3] Schurig D., Mock J., Justice B. J. (2006). Metamaterial electromagnetic cloak at microwave frequencies. Science.

[4] Zheng G., Mühlenbernd H., Kenney M., Li G., Zentgraf T., Zhang S. (2015). Metasurface holograms reaching 80% efficiency. Nat. Nanotechnol..

[5] Ni X., Kildishev A. V., Shalaev V. M. (2013). Metasurface holograms for visible light. Nat. Commun..

[6] Li J., Kamin S., Zheng G., Neubrech F., Zhang S., Liu N. (2018). Addressable metasurfaces for dynamic holography and optical information encryption. Sci. Adv..

[7] Luo X. Y., Guo W. L., Chen K. (2020). Active cylindrical metasurface with spatial reconfigurability for tunable backward scattering reduction. IEEE Trans. Antennas Propag..

[8] Chen K., Feng Y., Monticone F. (2017). A reconfigurable active Huygens’ metalens. Adv. Mater..

[9] Buchnev O., Podoliak N., Kaczmarek M., Zheludev N. I., Fedotov V. A. (2015). Electrically controlled nanostructured metasurface loaded with liquid crystal: toward multifunctional photonic switch. Adv. Opt. Mater..

[10] Yang W., Chen K., Zheng Y. (2021). Angular-adaptive reconfigurable spin-locked metasurface retroreflector. Adv. Sci..

[11] Zheng Y., Chen K., Yang W. (2022). Kirigami reconfigurable gradient metasurface. Adv. Funct. Mater..

[12] Yu N., Genevet P., Kats M. A. (2011). Light propagation with phase discontinuities: generalized laws of reflection and refraction. Science.

[13] Wang Y., Xu S., Yang F., Werner D. H. (2021). 1 bit dual-linear polarized reconfigurable transmitarray antenna using asymmetric dipole elements with parasitic bypass dipoles. IEEE Trans. Antennas Propag..

[14] Huang C., Zhang C., Yang J., Sun B., Zhao B., Luo X. (2017). Reconfigurable metasurface for multifunctional control of electromagnetic waves. Adv. Opt. Mater..

[15] Jiang L., Jing Y., Li Y. (2021). Multidimensionally manipulated active coding metasurface by merging pancharatnam–berry phase and dynamic phase. Adv. Opt. Mater..

[16] Qu G., Yang W., Song Q. (2020). Reprogrammable meta-hologram for optical encryption. Nat. Commun..

[17] Zhang L., Chen M. Z., Tang W. (2021). A wireless communication scheme based on space- and frequency-division multiplexing using digital metasurfaces. Nat. Electron..

[18] Tian S., Zhang X., Wang X., Han J., Li L. (2022). Recent advances in metamaterials for simultaneous wireless information and power transmission. Nanophotonics.

[19] Wang C., Xu H. X., Wang Y., Hu G., Luo H., Wang K. (2023). Reconfigurable transmissive metasurface synergizing dynamic and geometric phase for versatile polarization and wavefront manipulations. Mater. Des..

[20] Li L., Zhao H., Liu C., Cui T. J. (2022). Intelligent metasurfaces: control, communication and computing. eLight.

[21] Di Renzo M., Zappone A., Debbah M. (2020). Smart radio environments empowered by reconfigurable intelligent surfaces: how it works, state of research, and the road ahead. IEEE J. Sel. Areas Commun..

[22] Huang C. X., Zhang J., Cheng Q., Cui T. J. (2021). Polarization modulation for wireless communications based on metasurfaces. Adv. Funct. Mater..

[23] Zhang S., Zhang H., Di B. (2022). Intelligent omni-surfaces: ubiquitous wireless transmission by reflective-refractive metasurfaces. IEEE Trans. Wirel. Commun..

[24] Hu J., Zhang H., Di B. (2020). Reconfigurable intelligent surface based RF sensing: design, optimization, and implementation. IEEE J. Sel. Areas Commun..

[25] Zhao H., Shuang Y., Wei M., Cui T. J., Hougne P. D., Li L. (2020). Metasurface-assisted massive backscatter wireless communication with commodity Wi-Fi signals. Nat. Commun..

[26] Zhao J., Yang X., Dai J. Y. (2019). Programmable time-domain digital-coding metasurface for non-linear harmonic manipulation and new wireless communication systems. Natl. Sci. Rev..

[27] Dai J. Y., Tang W. K., Yang L. X. (2020). Realization of multi-modulation schemes for wireless communication by time-domain digital coding metasurface. IEEE Trans. Antennas Propag..

[28] Wu Q., Zhang R. (2019). Towards smart and reconfigurable environment: intelligent reflecting surface aided wireless network. IEEE Commun. Mag..

[29] Tennant A., Chambers B. (2009). Time-switched array analysis of phase-switched screens. IEEE Trans. Antennas Propag..

[30] Zhang N., Chen K., Zheng Y. (2020). Programmable coding metasurface for dual-band independent real-time beam control. IEEE J. Emerg. Sel. Top. Circuits Syst..

[31] You Y., Lu Y., You Q., Wang Y., Huang J., Lancaster M. J. (2018). Millimeter-wave high-gain frequency-scanned antenna based on waveguide continuous transverse stubs. IEEE Trans. Antennas Propag..

[32] Lv Q., Jin C., Zhang B., Shen Z. (2020). Frequency-scanning multipolarization antennas. IEEE Trans. Antennas Propag..

[33] Guo J., Wang T., Zhao H. (2019). Reconfigurable terahertz metasurface pure phase holograms. Adv. Opt. Mater..

[34] Cong L., Singh R. (2020). Spatiotemporal dielectric metasurfaces for unidirectional propagation and reconfigurable steering of terahertz beams. Adv. Mater..

[35] Hu Y., Tong M., Xu Z., Cheng X., Jiang T. (2021). Spatiotemporal terahertz metasurfaces for ultrafast all-optical switching with electric-triggered bistability. Laser Photonics Rev..

