# Supplementary material for: Broadband wireless communication with space-time-varying polarization-converting metasurface
Source: Nanophotonics. 2023 Mar 7;12(7):1327–36. doi: 10.1515/nanoph-2023-0027 (PMC11636446; doi:10.1515/nanoph-2023-0027)
Supplement: Supplementary file 1 — Supplementary Material Details [file j_nanoph-2023-0027_suppl.pdf]

Supporting Information

Broadband wireless communication with space-time-varying polarization-converting metasurface

Qi Hu, Ke Chen*, Yilin Zheng, Zhiyuan Xu, Jianmin Zhao, Jian Wang*, and Yijun Feng*

School of Electronic Science and Engineering, Nanjing University, Nanjing, 210093, China

*Corresponding author:
ke.chen@nju.edu.cn (Ke Chen);
wangjnju@nju.edu.cn (Jian Wang);
yjfeng@nju.edu.cn (Yijun Feng)

1. Design details of the proposed meta-atom

The current distributions of the meta-atom operating as "R_0" and "R_1" under an *x*-polarized incidence at 4.4 GHz is illustrated in Figure S1(a)-(b), respectively. For a clear view, we mark the switched-on diodes and leave out the switched-off diodes. It is observed that the induced currents are more concentrated on the gaps where the diodes are switched on instead of gaps where the diodes are switched off. Besides, the two different operation states exhibit almost mirror symmetric current distributions along *x*-axis.

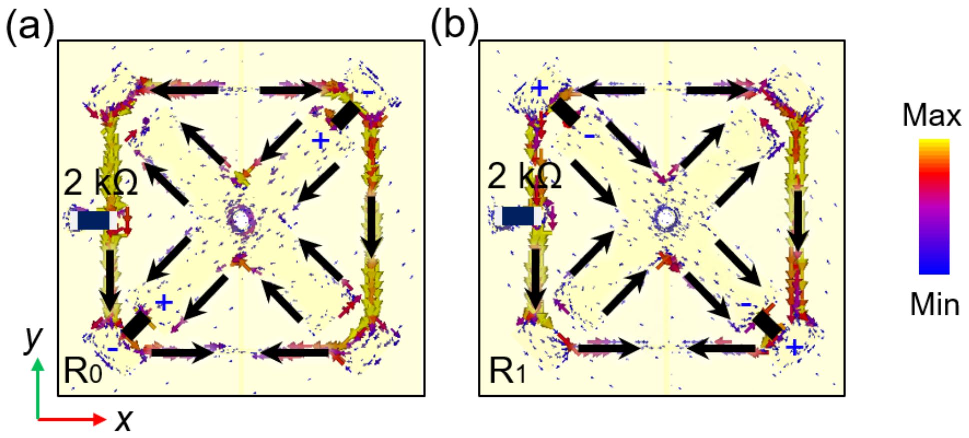

Supplementary Figure S1: The current distribution of the meta-atom at 4.4 GHz under an *x*-polarized incidence. (a) R_0. (b) R_1.

The simulated co-polarized reflection coefficients of the proposed meta-atom are plotted in Figure S2. It is observed that the two distinct operation states can exhibit almost identical reflection amplitude, which is suppressed below -10 dB within the whole operation bandwidth and below -28 dB at minimum.

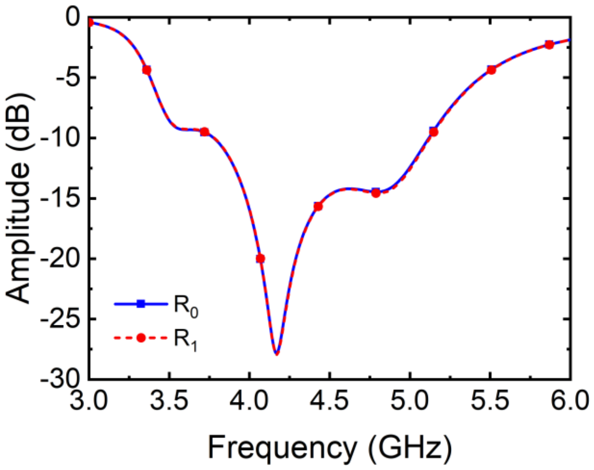

Supplementary Figure S2: Simulated co-polarized reflection amplitude of the proposed meta-atom.

2. Wireless coverage for three users

By adopting optimization algorithm, symbol “0” and “1” are mapping to spatial coding patterns of “$R_0R_0R_0R_0R_0R_0R_0R_0R_0R_0R_0R_0R_1R_1R_1R_1R_1R_1$…” and “$R_1R_1R_1R_1R_1R_1R_1R_1R_1R_1R_1R_1R_0R_0R_0R_0R_0R_0$…” to simultaneously provide wireless coverage for three users, respectively. As plotted in Figure S3(a)-(b), the simulated far-field scatterings under aforementioned coding patterns consist of main beams with identical elevation angles of 0° and ± 15° at 4.3 GHz，but distinguished by a 180° phase difference, thus satisfying the requirement for information transmission.

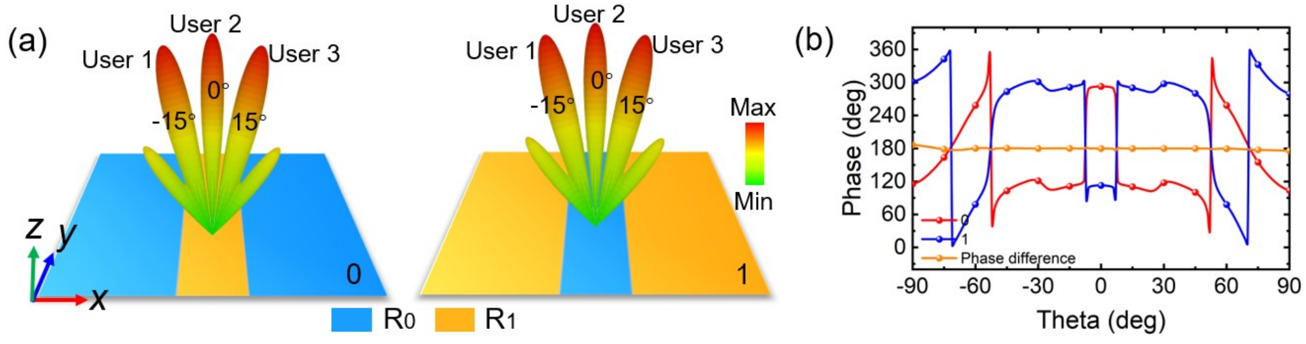

Supplementary Figure S3: Simulated far-field results.

Simulated (a) far-field scatterings and (b) phase difference between symbol “0” and “1”, which are corresponding to spatial coding patterns of “$R_0R_0R_0R_0R_0R_0R_0R_0R_0R_0R_0R_0R_1R_1R_1$ $R_1R_1R_1$…” and “$R_1R_1R_1R_1R_1R_1R_1R_1R_1R_1R_1R_1R_0R_0R_0R_0R_0R_0$…”, respectively.

3. Wireless communication adopting amplitude modulation scheme

To implement wireless communication adopting amplitude shift keying modulation scheme, the metasurface should provide at least two different reflection amplitudes. Here, we introduce another operation state “R_2” of the meta-atom by switching off the four PIN diodes to provide another reflection amplitude. The co-polarized reflection properties of the meta-atom operating as “R_0” and “R_2” are plotted in Figure S4(a), from which we can observe a near-zero reflection and a near-unity reflection, respectively. Therefore, a binary amplitude shift keying (BASK) scheme can be demonstrated by mapping binary symbols to two different far-field scatterings obtained by switching every meta-atom to “R_0” or “R_2” states, as shown in Figure S4(b). A good consistence can be observed between the to-be-transmitted picture (Figure S4(c)) and the recovered picture (Figure S4(d)). The measured constellation diagram displayed in Figure S4(e) conforms well to the ideal diagram.

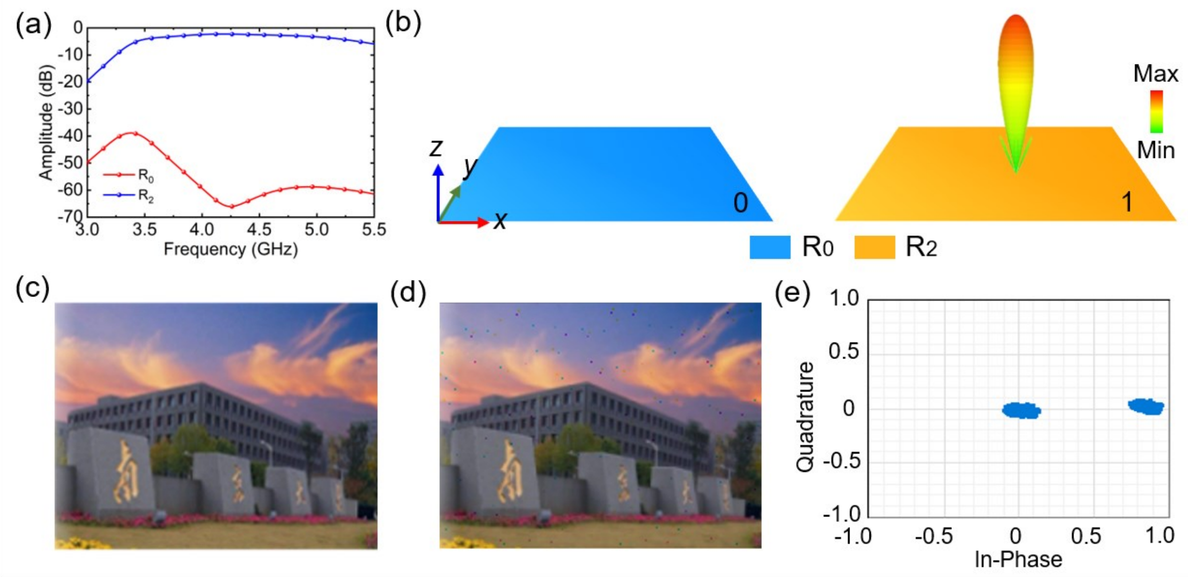

Supplementary Figure S4: Experimental demonstration of wireless communication adopting BASK scheme.

(a) Simulated co-polarized reflection of the meta-atom operating as “R_0” and “R_2”. (b) Simulated far-field scatterings by switching every meta-atom to “R_0” and “R_2” states. (c) To-be-transmitted color picture. (d) Recovered color picture. (e) Measured constellation diagram.

4. Simulated and measured far-field results

By fixing the mapping relationships between the binary symbols “0” and “1” and the spatial patterns to “$R_0R_0R_0R_0R_0R_1R_1R_1R_1R_1$…” and “$R_1R_1R_1R_1R_1R_0R_0R_0R_0R_0$…”, the simulated far-field patterns under the modulation of “$R_0R_0R_0R_0R_0R_1R_1R_1R_1R_1$…” (symbol “0”) at 3.7 GHz, 4.1 GHz, 4.6 GHz, and 5.1 GHz are plotted in Figure S5 as illustrative examples. It is observed that the incidence is anomalously deflected to twin beams pointing at ± 40°, ± 36°, ± 30°, and ± 28°, respectively, which are in consistence with theoretical predictions. The corresponding measured far-field patterns are plotted in Figure S6, from which we can observe the twin beams are approximately pointed to ± 40°, ± 36°, ± 30°, and ± 28° at different frequencies, verifying the potentials of the proposed system in frequency-scanning antennas.

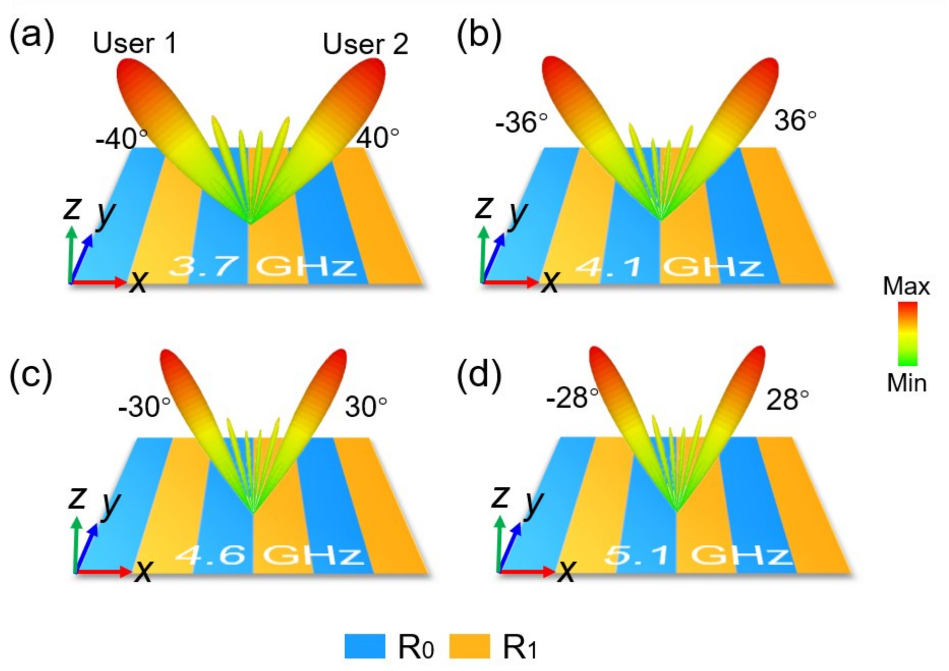

Supplementary Figure S5: Simulated scattering patterns under the modulation pattern of “$R_0R_0R_0R_0R_0R_1R_1R_1R_1R_1$…” (symbol “0”). (a) 3.7 GHz. (b) 4.1 GHz. (c) 4.6 GHz. (d) 5.1 GHz.

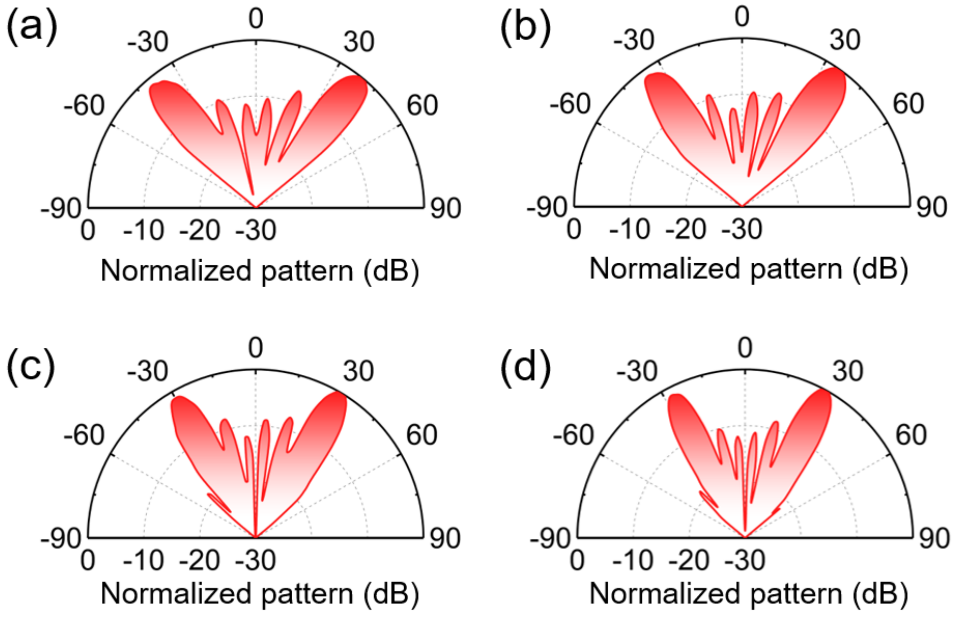

Supplementary Figure S6: Measured scattering patterns under the modulation pattern of "$R_0R_0R_0R_0R_0R_1R_1R_1R_1R_1$…" (symbol "0"). (a) 3.7 GHz. (b) 4.1 GHz. (c) 4.6 GHz. (d) 5.1 GHz.
